# Screening the Antibiotic Activity of Cave Actinobacteria Against Multidrug‐Resistant Strains of *Pseudomonas aeruginosa* and Methicillin‐Resistant *Staphylococcus aureus*

**DOI:** 10.1155/ijm/9984546

**Published:** 2026-02-05

**Authors:** Rajani Balkrishna Rao, Katelyn Boase, Cornelia Wuchter, Clem Kuek, Kliti Grice, Marco J. L. Coolen

**Affiliations:** ^1^ Curtin Malaysia Research Institute (CMRI), Curtin University, Miri, Sarawak, Malaysia, curtin.edu.au; ^2^ Western Australia Organic and Isotope Geochemistry Centre, School of Earth and Planetary Sciences, Curtin University, Perth, Western Australia, Australia, curtin.edu.au

**Keywords:** Actinobacteria, agar well diffusion, cross-streak method, multidrug-resistant bacteria, Mulu Caves

## Abstract

Many antibiotics originate from soil‐inhabiting Actinobacteria, especially from the diverse genus *Streptomyces*. However, the emergence of antibiotic resistance poses a significant global challenge to treating infectious diseases. Therefore, the search for Actinobacteria, particularly from less‐explored environments, as potential sources of novel antimicrobial compounds, is of great importance. This study sampled various biofilms growing on cave structures within Deer Cave and Lagang Cave, located in the UNESCO World Heritage Site of Gunung Mulu National Park (GMNP; Sarawak, Malaysia). From this relatively untapped niche in caves, we identified and screened actinobacterial isolates for their potential antimicrobial properties against drug‐resistant *Pseudomonas aeruginosa* and *Staphylococcus aureus* strains. Of 48 isolates, 24 showed inhibition of one or both drug‐resistant strains in the antimicrobial assays conducted using cross‐streak and agar well diffusion methods. The ethyl acetate extracts containing potential secondary metabolites demonstrated effective inhibition, particularly against the drug‐resistant Gram‐negative *P. aeruginosa*. In contrast, the supernatants obtained from aerobic cultivation exhibited comparatively better activity against Gram‐positive *S. aureus* strains. 16S rRNA gene sequencing analysis of the isolates revealed that all except one isolate belonged to the genus *Streptomyces*. Maximum likelihood bootstrap tree analysis strongly supported the correct clustering of the *Streptomyces* isolates with well‐known bioactive compound producers, such as *S. gardneri*, *S. laurentii*, and *S. zaomyceticus*. Notably, Deer Cave Isolate D3‐12 exhibited inhibitory activity against both drug‐resistant strains and, therefore, represents a promising candidate for future studies involving the characterization of its bioactive compounds. The remaining actinobacterial isolate exhibited 100% sequence homology to soil‐inhabiting *Rhodococcus pedecola*, known for its antibacterial properties. These findings suggest that the caves of GMNP harbor untapped ecological niches of diverse cave‐dwelling Actinobacteria, which may serve as sources of antimicrobial compounds effective against emerging antibiotic‐resistant pathogens.

## 1. Introduction

Infectious diseases lead to high morbidity and mortality in communities when not properly treated. Antimicrobial resistance ranks among the top 10 global public health threats [1]. Hospital environments host numerous drug‐resistant pathogens that cause hospital‐acquired infections, which often prolong recovery, increase costs, and elevate the risk of death. For instance, nearly two million people in the United States suffer from hospital‐acquired infections, resulting in 90,000 deaths annually [2]. Superbugs or multidrug‐resistant (MDR) bacteria carry multiple mutations that confer resistance to various antibiotic families [3]. With MDR bacteria on the rise, the search for novel antimicrobial compounds that are safe and effective against such pathogens is of dire necessity. Actinobacteria are recognized as the leading producers of several beneficial natural antimicrobial compounds, including tetracycline, macrolides, chloramphenicol, nucleosides, and polyenes [4, 5]. Notably, members of the genus *Streptomyces* have demonstrated strong antimicrobial, anti‐inflammatory, or anticancer properties [6]. Actinobacteria are widely distributed, especially in soils. Still, many members of this phylum have yet to be discovered and screened from underexplored environments for their antimicrobial activities against MDR bacteria [7, 8]. Actinobacteria from cave habitats are particularly suitable for isolating and screening for novel antimicrobials due to their ability to express unique physiological and biochemical properties as survival strategies under extreme conditions prevailing in caves, such as darkness, oligotrophic conditions, and high mineral concentrations [9–13]. For example, actinobacterial isolates from soils in calcareous caves in Pakistan revealed promising activity against pathogens, including MDR *Salmonella* [14]. However, ecological niches in caves beyond soils, where Actinobacteria with antimicrobial properties may also exist, remain relatively undersampled.

**Figure 1:**
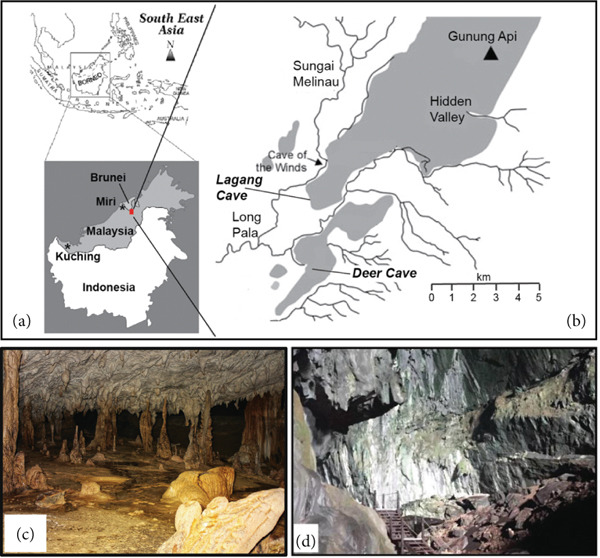
(a) Map of Southeast Asia with a zoom‐out of the island of Borneo (modified from [15]). The red box shows the location of the Gunung Mulu National Park (GMNP) in Sarawak, Malaysia. (b) Zoom‐in of the location of GMNP (modified from [16]). (c) Cave structures inside Lagang Cave (image by RBR). (d) Sun‐lit entrance of Deer Cave (image by MJLC).

This project is aimed at enriching and isolating Actinobacteria from biofilms overgrowing various cave structures in two previously unexplored cave systems, Deer Cave and Lagang Cave, located in Gunung Mulu National Park (GMNP) in Sarawak, Malaysia (Figure 1), and to screen these isolates for their antimicrobial potential to inhibit the growth of clinically relevant drug‐resistant pathogens. Serial dilution and selective media were used to enrich and isolate Actinobacteria from these understudied microbial niches in caves. The isolated actinobacterial colonies were subsequently screened for antimicrobial activity using the cross‐streak and agar well diffusion methods against clinically relevant, drug‐resistant Gram‐negative *Pseudomonas aeruginosa* and Gram‐positive *Staphylococcus aureus* strains. Finally, the taxonomic classification of the actinobacterial isolates was confirmed by 16S rRNA gene Sanger sequencing and placement in a maximum likelihood (ML) phylogenetic bootstrap tree alongside closely related reference sequences from Actinobacteria known for their antimicrobial properties.

## 2. Materials and Methods

### 2.1. Sampling Site and Sample Collection

This project was conducted under Biosecurity Permit No. (107) JHS/NCCD/600‐7/2/107/Jld.2, Park Permit No. WL59/2019, and R&D permit SBC‐2020‐RDP‐28‐MJLC, allowing us to conduct research in Sarawak. During a 3‐day sampling expedition to Deer Cave and Lagang Cave (Gunung Mulu National Park, Sarawak, Borneo, Malaysia; 4.056°N, 114.827°E) in April 2022, biofilms were sampled from four locations in Deer Cave and six locations in Lagang Cave (Figure 1, Figures S1 and S2). Biofilm samples were collected using sterile cotton swabs, resuspended in 1 mL of 0.9% sterile saline solution, kept in an Esky cooler filled with blue ice packs on‐site, and refrigerated at 4°C upon arrival at Curtin University, Malaysia.

### 2.2. Enrichment and Isolation of Actinobacterial Strains

One milliliter of each biofilm sample was serially diluted (10^−1^–10^−8^) in 0.9% sterile saline solution. Using the spread plate technique [17], 100 *μ*L of 10^1^–10^6^ times diluted samples was inoculated onto three different media: starch casein agar (SCA), actinomycete isolation agar (AIA), and International Streptomyces Project 4 (ISP4) agar (Himedia, Thane, India). All three media were supplemented with 75 *μ*g/mL nalidixic acid (MilliporeSigma, Burlington, Massachusetts, United States) and 50 *μ*g/mL cycloheximide (MilliporeSigma) to select for Actinobacteria and to inhibit Gram‐negative bacteria and fungi.

After inoculation, the plates were incubated for 14 days in an incubator (Binder KB240, Tuttlingen, Germany) at 28°C. The plates were examined daily for growth. Individual colonies with a filamentous, fungus‐like appearance typical for Actinobacteria, differing in texture, shape, and color, were selected and subcultured onto new plates of the same media and incubated at 28°C for 4–5 days until mature colonies developed. These mature colonies were inspected for pigmentation, aerial and substrate mycelia, and an earthy odor typical of Actinobacteria. Pure actinobacterial isolates were stored in 25% glycerol at −80°C at the Sarawak Biovalley Pilot Plant (Sarawak Research and Development Council) at Curtin University, Malaysia. These isolates are the property of the Sarawak Biodiversity Centre per the research permit (SBC‐2020‐RDP‐28‐MJLC).

### 2.3. Pathogens Used for Antimicrobial Screening

The antimicrobial activities of the actinobacterial isolates were screened against two pathogens: Gram‐negative *Pseudomonas aeruginosa* and Gram‐positive *Staphylococcus aureus*, using three strains of each. These included a laboratory strain, a hospital strain, and a drug‐resistant strain. The laboratory strains of *P. aeruginosa* (R. Hugh 813 NBRC 13275/ATCC 9027) and *S. aureus* (subspecies *aureus* Rosenbach NBRC 13276/ATCC 6538) obtained from the American Type Culture Collection (ATCC) have no known antibiotic resistance. *P. aeruginosa* BMH PA 01 and *S. aureus* BMH SA 01 represent hospital strains with unknown antibiotic susceptibilities that were isolated at the Borneo Medical Hospital (Miri, Sarawak) and donated for initial screening. The drug‐resistant *P. aeruginosa* (DRPA) (strain BAA‐2108) used in our study is resistant to imipenem, meropenem, and tobramycin and susceptible to aztreonam, cefepime, ceftazidime, ceftazidime–avibactam, ceftolozane–tazobactam, and piperacillin–tazobactam. The methicillin‐resistant *S. aureus* (MRSA) (strain BAA‐38) is furthermore resistant to oxacillin, cefoxitin, and tetracycline. Both strains were obtained from ATCC.

Strains obtained from ATCC were revived and maintained following the supplier′s instructions. The hospital strains were supplied on Luria–Bertani (LB) agar and subsequently cultured in LB broth (Merck) and on agar. These test cultures were prepared for subsequent antimicrobial screening by inoculating a single colony from the agar plate into 5 mL of sterile liquid broth. The test cultures were incubated at 37°C, with growth monitored by measuring the media′s optical density (OD) at 600 nm using a BioSpectrometer fluorescence (Eppendorf, Germany). Once the OD reached 0.1, these cultures were used for screening.

### 2.4. Screening of Actinobacterial Isolates for Antimicrobial Activities

#### 2.4.1. Primary Screening

Primary screening for the antimicrobial activities of the actinobacterial isolates was conducted using the cross‐streak method [18]. SCA, AIA, and ISP4 agar plates were streaked with individual actinomycete colonies, as shown in Figure S3a, and incubated at 28°C for 5 days in their respective growth media outlined above. On Day 5, 0.1 OD of the test cultures (laboratory strains of *P. aeruginosa* and *S. aureus* and hospital strains of *P. aeruginosa* and *S*. *aureus*, DRPA and MRSA) were streaked perpendicularly to the actinomycete isolates, and the plates were incubated at 37°C for 24 h (Figure S3a). The antibiotic activities of the actinobacterial isolates were expressed as the percentage of growth inhibition observed across the streaked pathogen line: undetermined growth inhibition (+/−) and no growth inhibition (−) vs. 25% (+), 50% (++), 75% (+++), and 100% growth inhibition (++++) (modified after [19]). Control plates without actinobacterial inoculation were prepared to monitor the expected growth of the test cultures on these media.

#### 2.4.2. Extraction of Secondary Metabolites

The actinobacterial isolates that showed inhibition against at least one *P. aeruginosa* or *S. aureus* strain in primary screening were grown using submerged aerobic cultivation to promote secondary metabolite production in liquid broth. Single colonies from these isolates were inoculated into sterile Erlenmeyer flasks containing 30 mL of sterile SCM, AIM, and ISP 4. The inoculated flasks were agitated at 180 rpm on a rotary incubator shaker (Innova 44R; Eppendorf, 1‐in. orbit diameter) and incubated for 5 days at 28°C. Afterwards, the culture media were separated into supernatant and cells by centrifugation (5804; Eppendorf) at 9600 × *g*. One hundred microliters of supernatant was analyzed for antimicrobial activity using the agar well diffusion method [18]. Equal volumes of methanol and ethyl acetate (Merck, Darmstadt, Germany) were added to the cell pellets and remaining supernatants, followed by overnight incubation at 28°C and agitation (180 rpm). The ethyl acetate extracts (i.e., the upper phase) were separated from the lower aqueous phase. The recovered methanol and ethyl acetate extracts were then tested for their antimicrobial activity using the agar well diffusion method as outlined in Figure S3b.

#### 2.4.3. Determination of Antimicrobial Activities

Mueller–Hinton agar (MHA) (Himedia) plates were swabbed with 0.1 OD of lab, hospital, and drug‐resistant strains of *P. aeruginosa* and *S. aureus*, which were cultured on their respective media, as mentioned above. After swabbing, the plates were kept at room temperature for 15 min. Eight‐millimeter‐sized wells were created using a sterile cork‐borer (Himedia) and inoculated with 100 *μ*L of supernatant, ethyl acetate crude extract, and methanol extract. The plates were incubated at 37°C for 24 h. The diameter of the zone of inhibition around the wells was measured for each actinomycete extract using a measuring scale [18]. If required, the inhibition zone around the wells of the actinomycete extract (mm) was corrected by subtracting the inhibition observed in the negative control wells (mm) incubated in parallel. Sterile media, ethyl acetate, and methanol were added to the wells as negative controls. Commercial antibiotic discs containing 10 *μ*g of gentamycin or 30 *μ*g of tetracycline were placed on the agar plates as positive controls to validate the assay (Oxoid‐ThermoFisher, Waltham, Massachusetts, United States) (Figure S3c).

#### 2.4.4. Characterization of Antibiotic‐Producing Actinobacterial Isolates

##### 2.4.4.1. Morphological Characterization

The actinobacterial isolates demonstrating effective inhibition were examined for their macroscopic features (colony texture, pigmentation color, and diffusion), morphological features (mycelium: aerial and substrate), and the presence of an earthy odor, typical of Actinobacteria [20, 21].

##### 2.4.4.2. Phylogenetic Characterization

Genomic DNA was extracted from the 5‐day‐old actinobacterial cultures using the DNeasy PowerBiofilm DNA extraction kit (Qiagen). A FastPrep‐96 instrument (MP Biomedicals, Santa Ana, California, United States) was set to 6.0 m/s for 20 s for the homogenization step. The genomic DNA extracts served as the template for subsequent 16S V3–V4 rRNA Sanger sequencing at the Australian Genome Research Facility (AGRF) in Perth, Australia. The obtained Sanger sequences were trimmed, and consensus sequences were created from aligned forward and reverse reads using Geneious 11.0.5 software [22]. Taxonomic identification of the ~400‐bp‐long consensus sequences based on closely related reference sequences in the RefSeq RNA database was performed using the Basic Local Alignment Search Tool for nucleotides (BLAST‐n) [23]. Using *Kitasatospora* as an outgroup, the unknown samples and their closest hits from BLAST‐n were aligned with MAFFT (Version 7.5) [24]. IQTREE 1.6.12 [25] was used to build a ML tree with the substitution model selected by IQTREE and 1000 bootstraps selected. Tree visualization was achieved using iTOL Version 6.8.1 [26].

#### 2.4.5. Nucleotide Sequence Accession Numbers

The 16S rRNA gene sequences of the 21 actinobacterial isolates supporting the findings are available in GenBank under accession numbers PQ614116–PQ614137, following an embargo from the date of publication to allow for commercialization of research findings.

## 3. Results

### 3.1. Enrichment and Isolation of Actinobacteria

**Figure 2:**
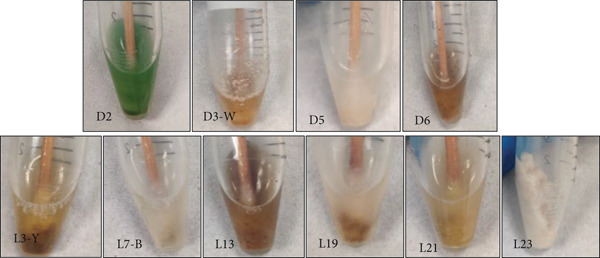
Biofilm samples collected from Deer Cave (D‐2, D‐3W, D‐5, and D‐6) and Lagang Cave (L‐3Y, L‐7, L‐13, L‐19, L‐21, and L‐23) in sterile 1 mL of 0.9% saline.

**Figure 3:**
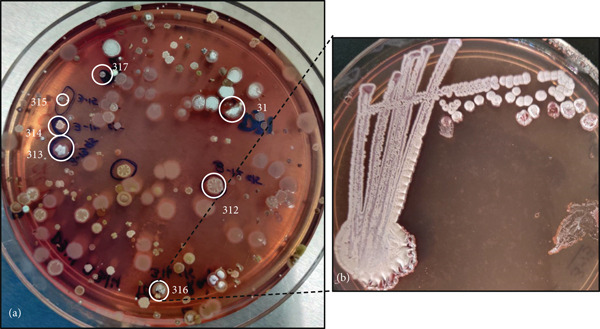
Representative images of an (a) SCA plate (primary isolation) showing encircled colonies of putative Actinobacteria selected for pure culture isolation from Location 3 in Deer Cave and (b) quadrant streaked Isolate D3‐16 selected from the SCA plate of panel (a).

Biofilm swab samples were collected from two different cave systems, Deer Cave and Lagang Cave (Figure 2), from 10 sampling sites (Figure S2). Following inoculation, distinct actinobacterial colonies appeared within 3–4 days of incubation. Sixty‐two actinobacterial colonies were initially selected from 10 biofilm samples to obtain individual colonies. However, 14 actinobacterial colonies could not be revived when subcultured onto new plates. Therefore, only 48 isolates were retrieved as single colonies/pure cultures (Figure 3).

### 3.2. Primary Screening

**Figure 4:**
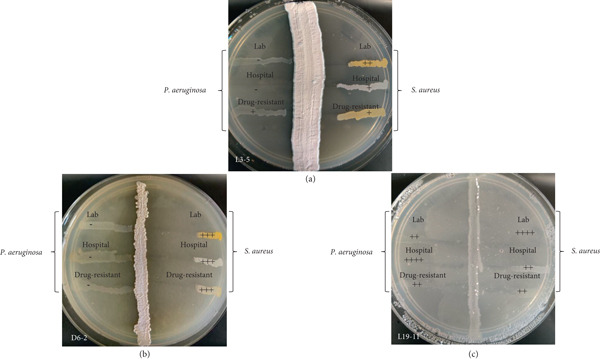
Screening of selected antibacterial isolates (a) L3‐5, (b) D6‐2, and (c) L19‐11 for their antagonistic activity using the cross‐streak method against *P. aeruginosa* and/or *S. aureus* test strains. Level of inhibition: no inhibition (–), 25% inhibition (+), 50% inhibition (++), 75% inhibition (+++), and 100% inhibition (++++). Refer to primary screening results for further details.

All 48 actinobacterial isolates were tested for their antibacterial activity against the *P. aeruginosa* and *S. aureus* strains. Fifty percent of the actinobacterial isolates (24 out of 48) showed antibacterial activity against at least one *P. aeruginosa* or *S. aureus* strain (Table 1). Twenty‐two isolates demonstrated antagonistic activity against at least one *S. aureus* strain, and 12 showed activity against at least one *P. aeruginosa* strain. Figure 4 displays the cross‐streak plates of three selected actinobacterial isolates: Isolate L3‐5 (Lagang Cave) did not show antagonistic activity against lab and hospital strains of *P. aeruginosa*, while it showed < 25% inhibition against the DRPA strain, ~50% inhibition against the lab strain of *S. aureus*, and ~25% inhibition against the hospital and drug‐resistant strains of *S. aureus*. Isolate 6‐2 (Deer Cave) showed ~75% inhibition of all three *S. aureus* strains. Isolate 19‐11 (Lagang Cave) showed ~50% inhibition of lab and DRPA strains as well as hospital and drug‐resistant strains of *S. aureus*, with complete inhibition of the hospital strain of *P. aeruginosa* and the lab strain of *S. aureus.*

**Table 1:** Primary screening of 24 actinomycetes isolates that showed inhibition against at least one *P. aeruginosa* or *S. aureus* strain and were selected for the agar well diffusion method.

**Cave**	**Isolate**	**Type of test organism**					
		***Staphylococcus aureus* strain**			***Pseudomonas aeruginosa* strain**		
		**Lab**	**Hospital**	**Drug resistant**	**Lab**	**Hospital**	**Drug resistant**
Deer	D3‐2	−	−	−/+	+	+	+
	D3‐3	+++	++	++	−	−	−
	D3‐7	−	−	++	−	−	−
	D3‐12	++++	+++	++++	−	−	−
	D3‐13	+	+	+	−	−	−
	D3‐14	+	−	+	−	−	−
	D3‐19	−	−	−	++++	−	++
	D3‐21	++	++	++	−	−	−
	D5‐9	++	−	−	+	+	+
	D6‐1	++	++	++	+	+	++
	D6‐2	+++	+++	+++	−	−	−
	D6‐3	+	−	−	−	−	−
	D6‐5	++	++	++	−	++	+++
	D6‐6	++++	++++	++++	++++	++++	++++

Lagang	L3‐2	++	++	+++	+	−	+
	L3‐3	+	−/+	−	−	−	−
	L3‐5	++	+	+	−	−	+
	L13‐3	+	+	++	−	+	+
	L19‐3	++++	−	++++	−	−	−
	L19‐4	−	−	−	−	+++	−
	L19‐11	++	++	++	++	++	+
	L23‐3	+	−	−	−	−	−
	L23‐5	++	+	+	−	−	−

Abbreviations: −, no inhibition; +, 25% inhibition or less; ++, 50% inhibition; +++, 75% inhibition; ++++, 100% inhibition; −/+, may or may not show inhibition.

### 3.3. Agar Well Diffusion

Agar well diffusion showed that all 24 actinobacterial isolates displayed antimicrobial activity against at least one strain of *P. aeruginosa* or *S. aureus*. The supernatant, ethyl acetate crude extract, and methanol extract were screened for antibiotic activity against both bacteria. The supernatant from six isolates demonstrated antimicrobial activity against *S. aureus* but not against *P. aeruginosa* (Table 2). The ethyl acetate crude extracts of all 24 actinobacterial isolates exhibited antimicrobial activity against *P. aeruginosa*, while *S. aureus* strains were most susceptible to the supernatant (Table 2). Methanol extracts showed negligible inhibition (1–2 mm) compared to supernatants and ethyl acetate extracts. While some strains revealed a larger zone of inhibition for the methanol extracts compared to the negative control, these differences were generally only marginal compared to the inhibition zones from the ethyl acetate crude extracts (Table 2). Hence, the methanol extracts were excluded from further analysis. Five actinobacterial isolates showed activity (i.e., a zone of inhibition of 2 mm or larger than the negative control) against at least one strain of *S. aureus* (Figure 5, Table 2). In contrast, all 24 isolates showed activity against at least one strain of *P. aeruginosa* (Figure 5, Table 2). Among all the isolates, D3‐12 showed the highest inhibitory activity against both drug‐resistant strains.

**Table 2:** Corrected zone of inhibition (mm) for the 24 actinobacterial isolates with antimicrobial activity against *P. aeruginosa* and *S. aureus* test strains.

**Cave**	**Isolates**	**Type of extract**	***Staphylococcus aureus***			***Pseudomonas aeruginosa***		
			**Lab**	**Hospital**	**Drug resistant**	**Lab**	**Hospital**	**Drug resistant**
Deer	D3‐2	Supernatant		4	6			
		Ethyl acetate			1	4		1
		Methanol						

Deer	D3‐3	Supernatant						
		Ethyl acetate			1	3		1
		Methanol						

Deer	D3‐7	Supernatant						
		Ethyl acetate				10		8
		Methanol			1		1	

Deer	D3‐12	Supernatant	9	9	2			
		Ethyl acetate	2	3	4	5		7
		Methanol			4		1	

Deer	D3‐13	Supernatant						
		Ethyl acetate				12	2	4
		Methanol						

Deer	D3‐14	Supernatant						
		Ethyl acetate				9	12	6
		Methanol				1		

Deer	D3‐19	Supernatant						
		Ethyl acetate		1		7	9	3
		Methanol						

Deer	D3‐21	Supernatant	4	3				
		Ethyl acetate	1			3	1	1
		Methanol				1		

Deer	D5‐9	Supernatant	1					
		Ethyl acetate				3	11	3
		Methanol						

Deer	D6‐1	Supernatant						
		Ethyl acetate					6	2
		Methanol						

Deer	D6‐2	Supernatant						
		Ethyl acetate			1		14	6
		Methanol						

Deer	D6‐3 (A)	Supernatant						
		Ethyl acetate				5	4	6
		Methanol					1	
Deer	D6‐5	Supernatant						
		Ethyl acetate			1	3	3	4
		Methanol		1				

Deer	D6‐6	Supernatant						
		Ethyl acetate				6	1	4
		Methanol		2				

Lagang	L3‐2	Supernatant	1					
		Ethyl acetate				4		2
		Methanol						

Lagang	L3‐3	Supernatant						
		Ethyl acetate			1	4	2	7
		Methanol						

Lagang	L3‐5	Supernatant						
		Ethyl acetate		1		2	4	8
		Methanol				2		

Lagang	L13‐3	Supernatant						
		Ethyl acetate					3	5
		Methanol						

Lagang	L19‐3	Supernatant	4	2	2			
		Ethyl acetate		1		10	2	1
		Methanol	1			1		

Lagang	L19‐4	Supernatant						
		Ethyl acetate				15		1
		Methanol						

Lagang	L19‐11	Supernatant						
		Ethyl acetate				6	1	1
		Methanol						

Lagang	L23‐3	Supernatant						
		Ethyl acetate				1	2	2
		Methanol					1	

Lagang	L23‐4	Supernatant						
		Ethyl acetate	4	4	3	1	11	3
		Methanol						

Lagang	L23‐5	Supernatant						
		Ethyl acetate				2	10	1
		Methanol					1	

Figure 5Representative image of antimicrobial activity screening using the agar well diffusion method showing the corrected zone of inhibition. A 2–3 mm zone of inhibition against MRSA was observed with (a) supernatant from Isolate D3‐12 and (b) ethyl acetate extract of Isolate L23‐4. (c) Ethyl acetate extracts from Isolates L3‐3, L3‐5, and L13‐3 resulted in inhibition zones of 7, 8, and 5 mm, respectively, against DRPA. NC, negative control; PC, positive control with a known concentration of antibiotic disc.

**Figure:**
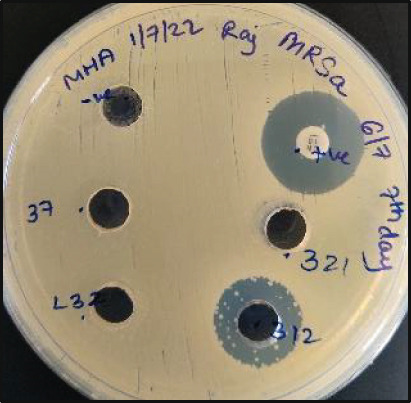
(a)

**Figure:**
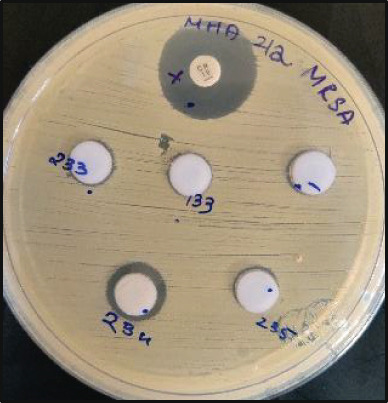
(b)

**Figure:**
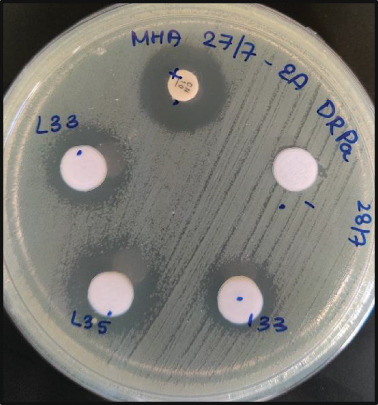
(c)

### 3.4. Characterization of Antibiotic‐Producing Actinobacterial Isolates

#### 3.4.1. Morphological Characterization

Actinobacterial isolates exhibiting antibiotic‐producing activity were examined for their macroscopic characteristics (growth, pigmentation color, and diffusion), morphological features (aerial and substrate mycelia), and an earthy odor, typical of Actinobacteria. See Table 3 for detailed descriptions of the 24 isolated antibiotic‐producing Actinobacteria.

**Table 3:** Morphological characterization of actinobacterial isolates grown as single colonies [20, 21].

**Cave type**	**Media**	**Isolate number**	**Description**					
			**Pigment diffusion**	**Pigment color**	**Smell**	**Aerial mycelium**	**Substrate mycelium**	**Colony characteristics**
Deer	AIA	D3‐2	N		N	Cream	Cream	Moist
	AIA	D3‐3	N		Y	Creamish pink	Yellowish pink	Dry flaky
	SCA	D3‐7	N		Undefined	Cream	Cream	Dry
	SCA	D3‐12	Y	Red	Y	White	Yellow	Floral shaped
	SCA	D3‐13	Y	Pinkish red	Y	White	Bright pink	Dry
	SCA	D3‐14	N		Undefined	Cream	Cream	Dry
	ISP4	D3‐19	Y	Yellowish pink	Y	White	Cream	Dry
	ISP4	D3‐21	N		Y	Pink brown orangish	Brown	Dry flaky
	AIA	D5‐9	N		Y	White	Cream brown	Powdery
	SCA	D6‐1	Y	Yellow	Y	Lemon yellow	Yellow	Powdery
	SCA	D6‐2	Y	Yellow	Y	Cream	Cream	Floral shaped
	AIA	D6‐3	N		Y	White	Yellow	Powdery
	ISP4	D6‐5	N		Y	Grey white	Brown	Dry
	ISP4	D6‐6	N		Y	Grey	Yellow	Dry

Lagang	SCA	L3‐2	Y	Pink	Y	Whitish yellow	Yellowish brown	Powdery
	ISP4	L3‐3	Y	Brown	Y	Grey	Brown	Dry
	ISP4	L3‐5	N		Y	Light pink	Brown	Powdery
	SCA	L13‐3	N		Undefined	Whitish green	Cream	Dry flaky
	ISP4	L19‐3	Y	Brown	Y	Grey	Creamish brown	Dry
	ISP4	L19‐4	N		Y	Pink	Cream	Dry flaky
	ISP4	L19‐11	N		Y	White brown	Cream	Dry
	AIA	L23‐1	Y	Brown	Y	Yellowish cream	Yellowish cream	Floral shaped
	ISP4	L23‐3	Y	Brown	Y	Brown	Brown	Floral shaped
	ISP4	L23‐4	Y	Brown	Y	Brown	Brown	Floral shaped
	ISP4	L23‐5	Y	Brown	Y	Brown	Brown	Floral shaped

*Note:* Pigment diffusion—presence or absence of diffusion of pigment from isolates into the agar medium; pigment color—color of the agar medium following pigment diffusion; smell—presence/absence of the characteristic earthy smell associated with actinomycetes; aerial mycelium—color of the mature spore‐bearing hyphae observed on the surface (top side) of the media plate; substrate mycelium—color as viewed from the reverse (under) side of the mass growth on the media plate.

Abbreviation: N, no; Y, yes.

#### 3.4.2. Molecular Identification of Actinobacterial Isolates

**Figure 6:**
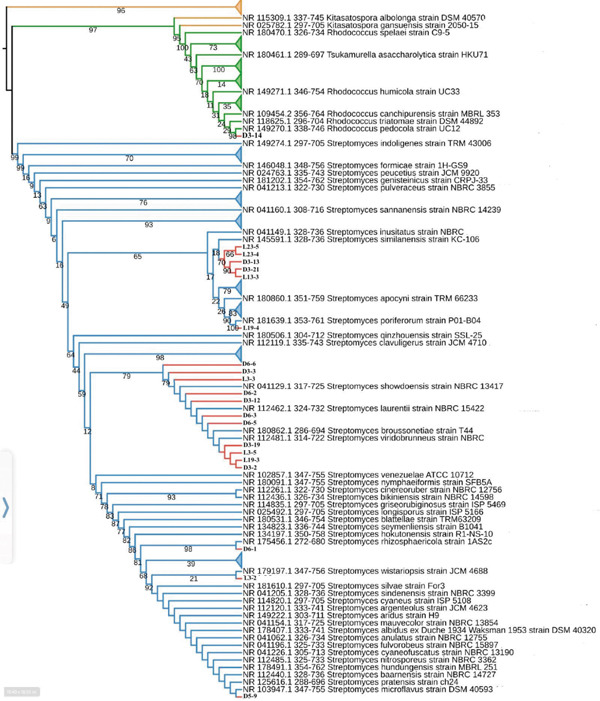
Maximum likelihood phylogenetic tree of partial 16S rRNA gene sequences (V3+V4 region) from 21 actinomycete strains isolated from the subsampled biofilms in Deer Cave and Lagang Cave (in bold) and their closest relatives present in NCBI′s RefSeq nr/nt database. A bootstrap replication of 1000x was used, and bootstrap values are shown at the respective branches.

Isolates D3‐7, L19‐11, and L23‐3 showed poor sequencing results and were excluded from further analysis. Using the ML method, a phylogenetic tree was constructed based on the 16S V3–V4 rRNA gene sequences of the remaining 21 isolates (denoted in bold), along with reference taxa of their closest environmental relatives retrieved from NCBI′s RefSeq database (Figure 6). Isolate D3‐14 demonstrated 100% sequence similarity with *Rhodococcus pedocola*, and its correct placement in the ML tree (Figure 6) was supported by a bootstrap value of 98. The other 20 isolates showed between 99.5% and 100% sequence similarity with various *Streptomyces* species. Isolates L23‐5, L23‐4, D3‐13, D3‐21, and D13‐3 grouped with *S. similanensis* and *S. inusitatus* with moderate support for their correct phylogenetic position in the ML tree (i.e., bootstrap value of 65) (Figure 6). L19‐4 was closely related to *S. poriferorum*, with a bootstrap value of 100, strongly supporting its correct phylogenetic placement. Isolate D3‐12, which showed the highest inhibitory activity against the DRPA and *S. aureus* strains, along with Isolates D3‐2, D3‐3, D3‐12, D3‐19, D6‐2, D6‐3, D6‐5, and D6‐6 from Deer Cave and Isolates L3‐3, L3‐5, and L19‐3 from Lagang Cave, clustered with four other *Streptomyces* reference sequences in the ML tree (*S. showdoensis* strain NBRC 13417, *S. laurentii* strain NBRC 15422, *S. broussonetiae* strain T44, and *S. viridobrunneus* strain NBRC 15902) with good support for their phylogenetic placement (bootstrap value of 79). Isolate D6‐1 clustered with *S. rhizospericola* strain 1AS2c with a bootstrap value of 97, suggesting strong support for their correct phylogenetic position in the ML tree. D5‐9 clustered with 15 other *Streptomyces* reference sequences, such as *S. silvae* strain Fo3, *S. sindenensis* strain NBRC 3399, *S. microflavus* strain DSM 40593, and *S. pratensis* strain ch24 (Figure 6). Finally, L3‐2 showed the highest sequence similarity with *S. wistariopsis* strain JCM 4688, but with poor support for its phylogenetic position in the ML tree (bootstrap value of 21) (Figure 6).

## 4. Discussion

The number of untreatable infections caused by MDR pathogens is rapidly increasing. One strategy to combat the spread of antibiotic resistance is to focus on screening undersampled ecological niches for the presence of Actinobacteria with antimicrobial activities against drug‐resistant clinical pathogens [8, 27]. In this study, we screened actinobacterial isolates from biofilms inside two unexplored cave systems (Deer and Lagang Caves) for their ability to inhibit the growth of DRPA (strain BAA‐2108) and *S. aureus* (MRSA strain BAA‐38). Twenty‐four of 48 isolates demonstrated antibacterial activity against at least one drug‐resistant test strain. Several isolates, such as D3‐12, L19‐3, and L23‐4 (Table 3), could inhibit both DRPA and MRSA. Among these three isolates, D3‐12 demonstrated the highest inhibitory activity against both drug‐resistant strains. The primary cross‐streak screening showed inhibition against MRSA but was less effective against DRPA. Unlike methanol extracts, the supernatant and ethyl acetate extracts exhibited inhibitory effects in the agar well diffusion assay. Notably, the ethyl acetate extracts from all 24 isolates inhibited DRPA to varying degrees. In contrast, inhibition of *S. aureus* strains was more effective using the supernatant than the ethyl acetate extract. Although it is difficult to determine the exact reason for these observations without further characterization of the bioactive compounds, the extraction efficiency of antimicrobial compounds is greatly affected by the type of solvent and its polarity [28]. Ethyl acetate, which is moderately polar, facilitates clear phase separation and concentration of secondary metabolites [29, 30]. In contrast, methanol is highly polar and primarily extracts proteins, sugars, and lipids, which are not usually classified as secondary metabolites [31]. In the case of the supernatant, the expression of these secondary metabolites without extraction and concentration might have occurred at concentrations below the detectable threshold [32, 33].

The inhibitory effect of the actinobacterial isolates from understudied cave biofilms against DRPA is a significant finding, as antibiotic compounds are generally more effective against Gram‐positive bacteria, such as *Staphylococcus*, which have a simpler cell wall structure than Gram‐negative bacteria, such as *Pseudomonas*. Specifically, Gram‐negative bacterial cell walls comprise a lipopolysaccharide barrier, making them more resistant and less susceptible to therapeutic drugs [34, 35]. According to the WHO, among the 60 products in development, very few specifically target Gram‐negative pathogens, highlighting the ongoing challenges in treating infections caused by this drug‐resistant bacterial group [36].

Identification of the 400 bp long sequences through 16S rRNA Sanger sequencing revealed that all but one isolate belonged to the genus *Streptomyces*. Members of this genus of Actinobacteria continue to produce most known compounds with antimicrobial, anti‐inflammatory, cytotoxic, and antitumor properties [37], and some *Streptomyces* isolated from underexplored cave biofilms in our study exhibited promising inhibition against drug‐resistant strains of *P. aeruginosa* and *S. aureus*. Bootstrap ML analysis showed medium to high support for the accurate classification of the *Streptomyces* isolates identified by 16S rRNA gene sequencing (e.g., D3‐12, which exhibited inhibition of both MDR *P. aeruginosa* and *S. aureus* strains) with well‐known bioactive substance–producing *Streptomyces* such as *S. gardneri*, *S. zaomyceticus*, *S. exfoliatus*, and *S. laurentii*. A strain of *S. gardneri* was found to produce a thiopeptide antibiotic that was effective against Gram‐positive aerobes and anaerobes [38]. *S. laurentii* is known to produce thiostrepton, a cyclic oligopeptide antibiotic of the thiopeptide class [39], and possesses antibacterial activity against *E. coli* and *B. cereus* [40]. *S. zaomyceticus* has shown good inhibitory activity against plant pathogens such as *Pseudomonas syringae* pv. *tomato*, *Fusarium oxysporum* f. sp. *momordicae*, and *Colletotrichum orbiculare* [41]. Exfoliamycin and its naphthoquinone derivatives extracted from *S. exfoliates* were shown to inhibit several Gram‐positive bacteria, such as *Bacillus subtilis*, *B. brevis*, and *Micrococcus luteus*. [42]. The sole isolate (D3‐14) that did not belong to *Streptomyces* showed 100% sequence homology to *Rhodococcus pedecola* strain UC12. Previously isolated from soil, strain UC12 has demonstrated antimicrobial properties against common pathogens, including *E. coli*, *S. aureus*, *B. subtilis*, and *P. aeruginosa* [43].

The media and cultivation conditions used in this study may have favored the growth of the rapidly growing actinobacterial genera, namely, *Streptomyces* (Streptomycetales) and *Rhodococcus* (Corynebacteriales). Since only a relatively small percentage of microbial community members from complex environmental samples can be cultivated, most escape isolation [44]. The analysis of metagenome‐assembled genomes (MAGs) in complex environmental samples, such as cave biofilms, can help identify the presence of fastidious actinobacterial taxa like *Acidothermus*, *Aeromicrobium*, *Actinomadura*, *Actinoplanes*, *Crossiella*, *Gaiella*, *Gordonia*, *Nocardioides*, *Pseudonocardia*, *Rubrobacter*, and *Solirubrobacter*, which may possess antimicrobial properties and have escaped cultivation, thereby guiding the development of new media tailored to their specialized nutritional needs [37, 45, 46]. A recent study, which advanced by including metabolomic analysis of extracted metabolites, revealed that most isolated actinobacterial strains from soils in undisturbed caves in Pakistan produced compounds active against several bacterial pathogens [47]. Together, these studies emphasize integrating screening techniques with additional omics approaches to provide more precise insights and improve the discovery of novel bioactive compounds.

Our study contributes to the growing recognition that caves provide a diverse range of microbial niches, extending beyond soils, and serve as promising, underexplored, and unique sources of bioactive compounds that may prove effective against emerging antibiotic‐resistant pathogens [48, 49].

## 5. Conclusions and Outlook

The understudied biofilms from the unexplored Deer and Lagang Caves in Gunung Mulu National Park are promising sources of antibiotic‐producing Actinobacteria capable of inhibiting the growth of MDR pathogens. The observed potent inhibitory effect on drug‐resistant *Pseudomonas aeruginosa* is relevant, as it targets Gram‐negative bacteria responsible for infectious diseases that are notoriously difficult to treat. Further studies could involve identifying and characterizing bioactive compounds, followed by their large‐scale production and testing their efficacy and toxicity against other drug‐resistant pathogens. Among the 24 isolates, D3‐12 appears to be the most promising due to its activity against both drug‐resistant strains and should be prioritized for further characterization. Subsequently, these efforts will help identify a single “lead” strain. Additionally, to ensure the isolation of rarer, non‐*Streptomyces* species from cave biofilms, future research could involve optimizing enrichment media, extending the incubation period, and implementing novel pretreatment methods. These approaches can be combined with culture‐independent metagenomics analysis (i.e., MAGs) to identify novel members of Actinobacteria and discover genes responsible for producing secondary metabolites within these caves.

## Conflicts of Interest

The authors declare no conflicts of interest.

## Author Contributions

Rajani Balkrishna Rao collected the samples, conducted the experiments, and led the manuscript writing. Katelyn Boase performed the bioinformatics and biostatistics analyses. Cornelia Wuchter assisted in data analysis and interpretation and contributed to the manuscript writing. Clem Kuek aided in sampling and contributed to the manuscript writing. Kliti Grice also contributed to the manuscript writing. Marco J.L. Coolen supported sampling, led the project supervision, and cowrote the manuscript with Rajani Balkrishna Rao.

## Funding

This study was funded by the Curtin Malaysia Research Institute (CMRI) Top‐Down PhD Grant, CMRI 6027.

## Supporting information


**Supporting Information** Additional supporting information can be found online in the Supporting Information section. Figure S1: Locations in (a) Deer Cave and (b) Lagang Cave where fresh biofilms were sampled for the isolation and screening of Actinobacteria for potential antibiotic production (cave map source: Department of Forestry, Sarawak). Figure S2: Biofilm samples collected from Deer Cave (D‐2, D‐3W, D‐5, and D‐6) and Lagang Cave (L‐3Y, L‐7, L‐13, L‐19, L‐21, and L‐23) within the Gunung Mulu National Park. Figure S3: Primary screening using the cross‐streak method. First, each actinobacterial isolate was streaked in the center of the plate. After 5 days of incubation at 28°C, the laboratory, hospital, and drug‐resistant strains of *S. aureus* and *P. aeruginosa* were streaked perpendicular to the grown actinobacterial isolates. The distance of inhibition on each side of the central streak was measured at the end of incubation. (b) Workflow after the submerged fermentation of the Actinobacteria. (c) Antimicrobial activity screening method of agar well diffusion using MHA plates with sample wells, negative control, and a commercial antibiotic disc as a positive control. Figure S4: Examples of media plates showing a zone of inhibition in negative control (NC) wells alongside the test samples. Although the agar well diameter was 8 mm, the negative controls (NCs) containing (a, b) ethyl acetate and (c) methanol showed slight inhibition of the test pathogens. Therefore, the “corrected zones of inhibition” reported in our study were calculated by subtracting the inhibition in the corresponding NC (mm) from that of the test sample (mm).

## Data Availability

The data that support the findings of this study are openly available in GenBank at https://ncbi.nlm.nih.gov.
