# Supplementary figures and images for: Screening the Antibiotic Activity of Cave Actinobacteria Against Multidrug‐Resistant Strains of Pseudomonas aeruginosa and Methicillin‐Resistant Staphylococcus aureus
Source: Int J Microbiol. 2026 Feb 5;2026:9984546. doi: 10.1155/ijm/9984546 (PMC12876465; doi:10.1155/ijm/9984546)

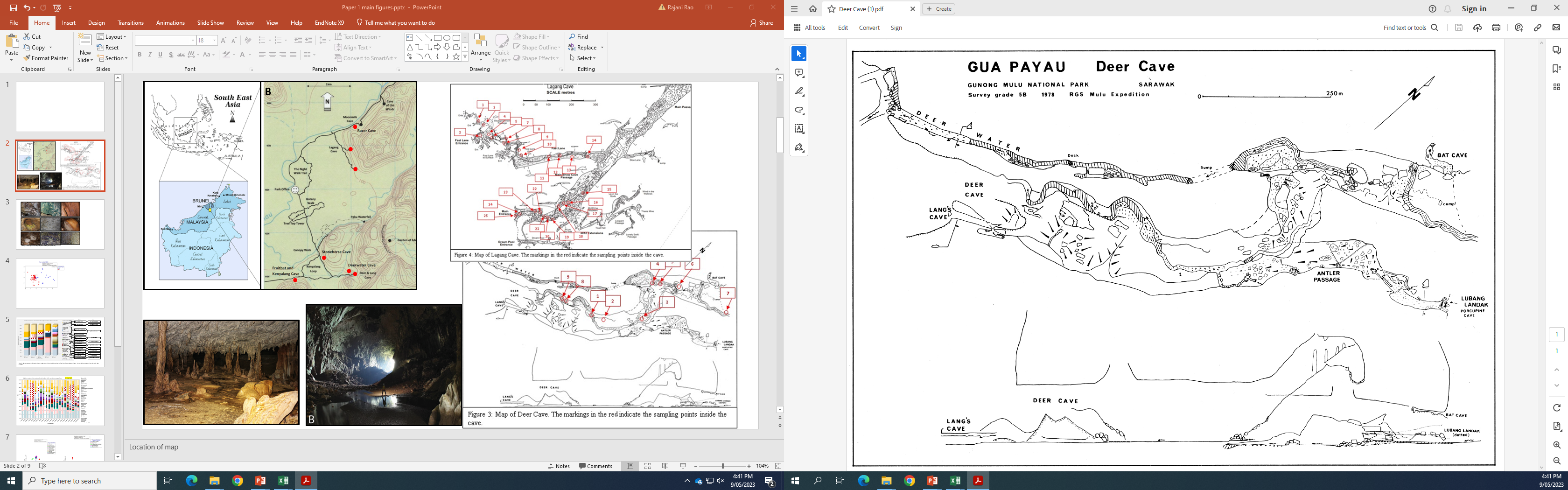


**2**

**3**

**5**

**6**


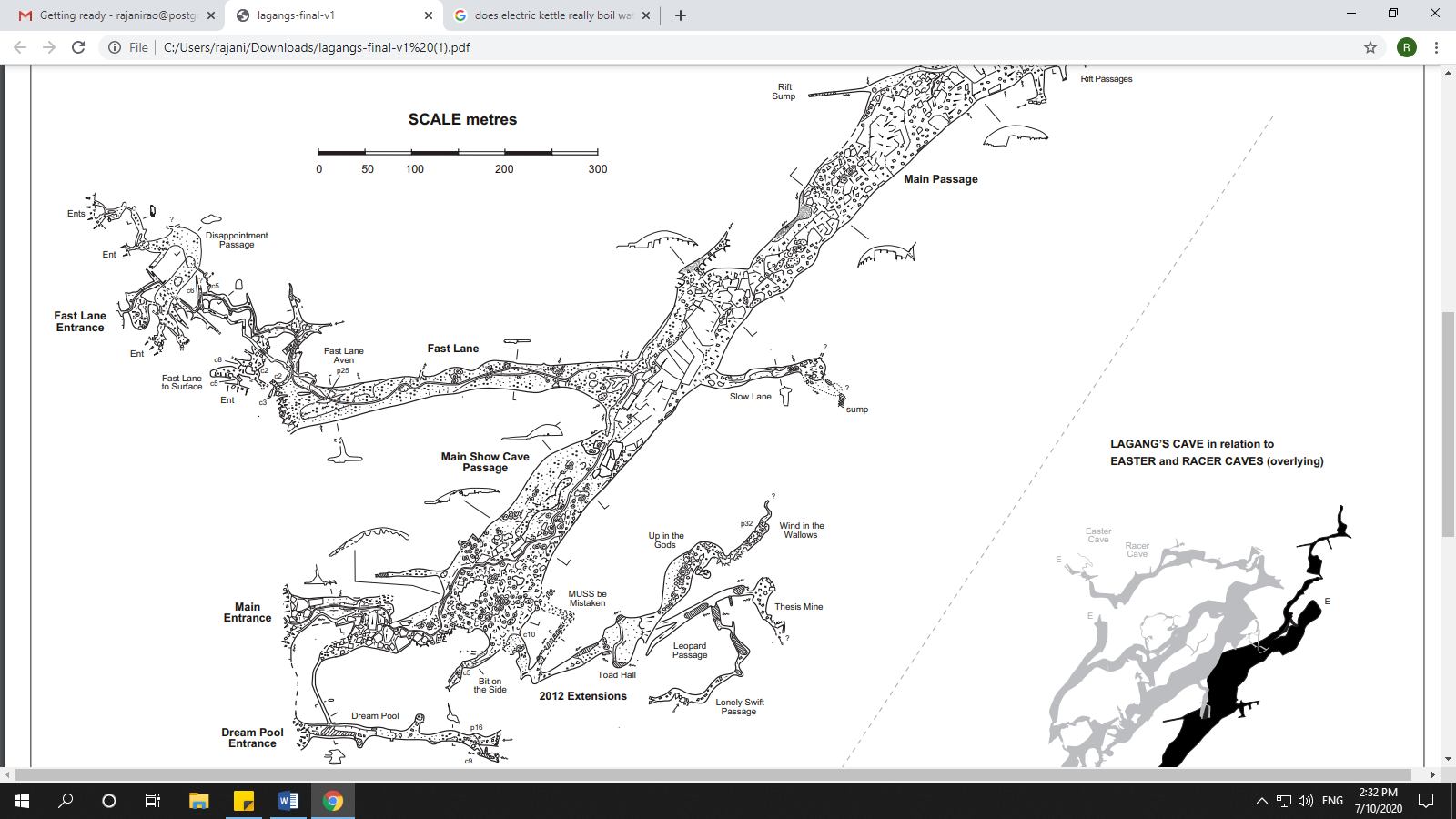


**3**

**7**

**13**

**19**

**21**

**23**

**Lagang Cave**

**a)**

**b)**

Supplement: Supplementary file 1 — Supporting Information Additional supporting information can be found online in the Supporting Information section. Figure S1: Locations in (a) Deer Cave and (b) Lagang Cave where fresh biofilms were sampled for the isolation and screening of Actinobacteria for potential antibiotic production (cave map source: Department of Forestry, Sarawak). Figure S2: Biofilm samples collected from Deer Cave (D‐2, D‐3W, D‐5, and D‐6) and Lagang Cave (L‐3Y, L‐7, L‐13, L‐19, L‐21, and L‐23) within the Gunung Mulu National Park. Figure S3: Primary screening using the cross‐streak method. First, each actinobacterial isolate was streaked in the center of the plate. After 5 days of incubation at 28°C, the laboratory, hospital, and drug‐resistant strains of S. aureus and P. aeruginosa were streaked perpendicular to the grown actinobacterial isolates. The distance of inhibition on each side of the central streak was measured at the end of incubation. (b) Workflow after the submerged fermentation of the Actinobacteria. (c) Antimicrobial activity screening method of agar well diffusion using MHA plates with sample wells, negative control, and a commercial antibiotic disc as a positive control. Figure S4: Examples of media plates showing a zone of inhibition in negative control (NC) wells alongside the test samples. Although the agar well diameter was 8 mm, the negative controls (NCs) containing (a, b) ethyl acetate and (c) methanol showed slight inhibition of the test pathogens. Therefore, the “corrected zones of inhibition” reported in our study were calculated by subtracting the inhibition in the corresponding NC (mm) from that of the test sample (mm). [file IJM-2026-9984546-s001.zip › Figure S1.docx]

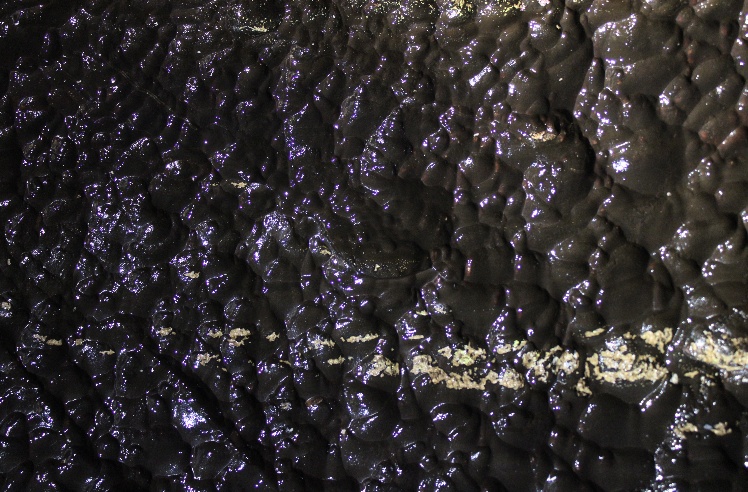

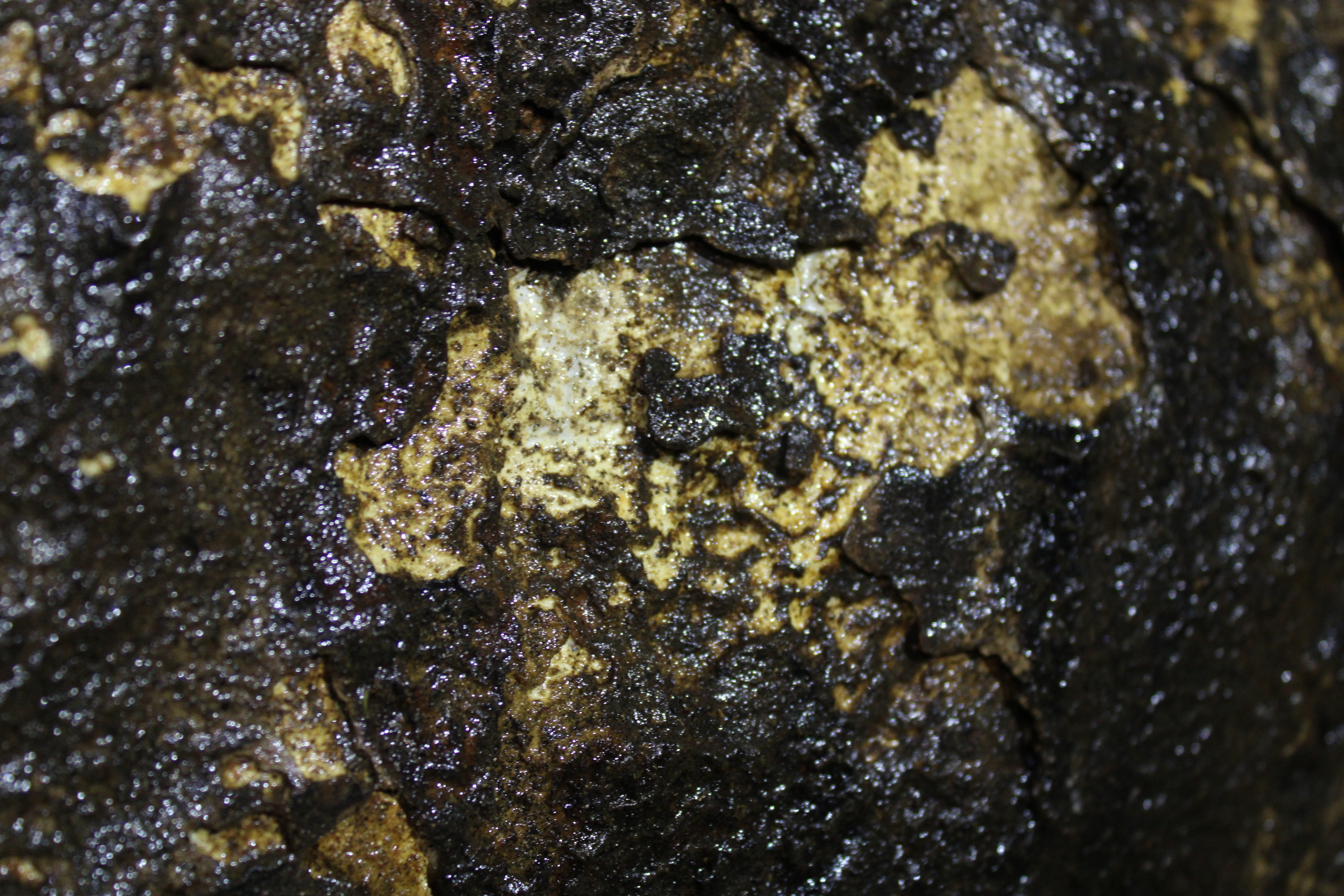

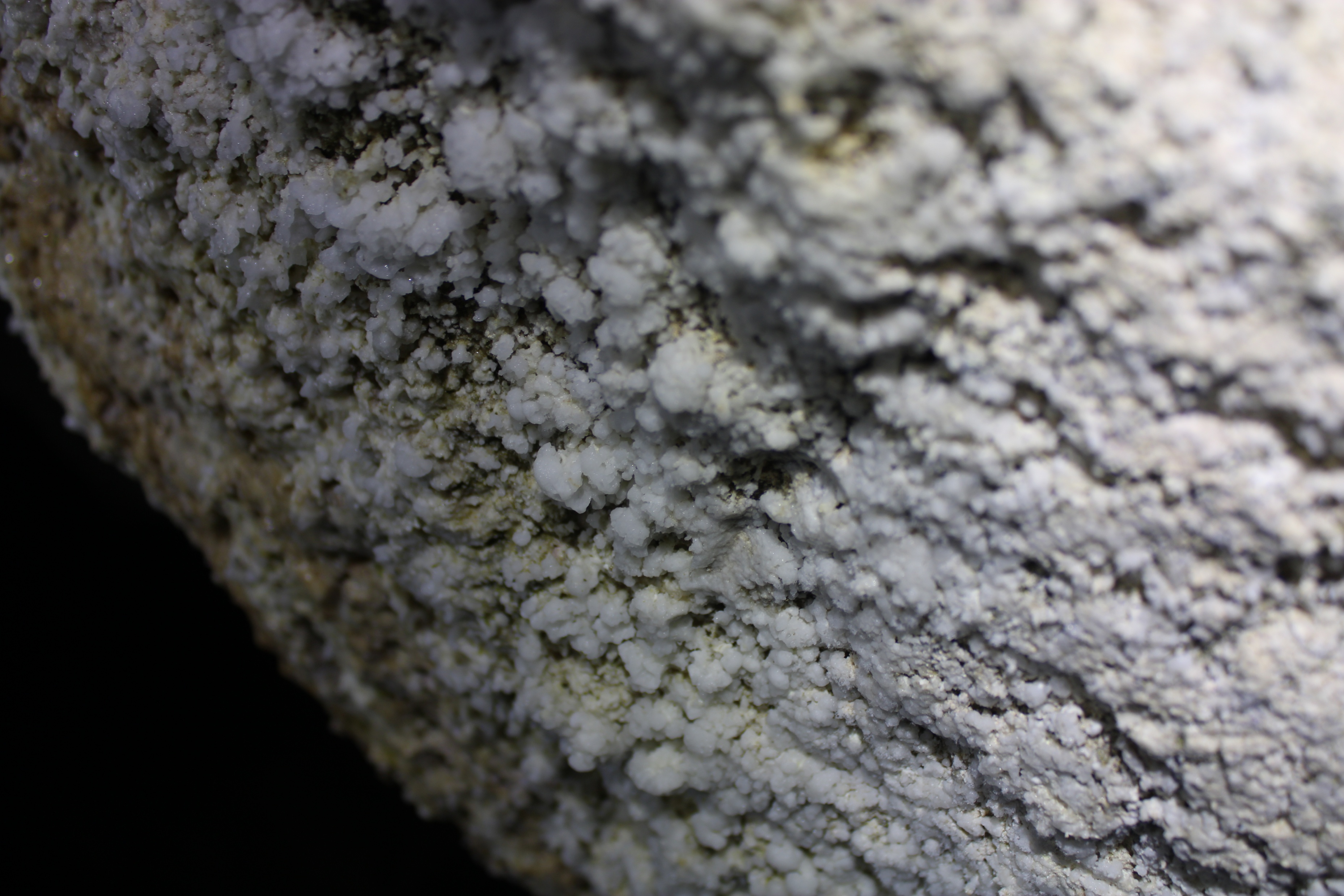

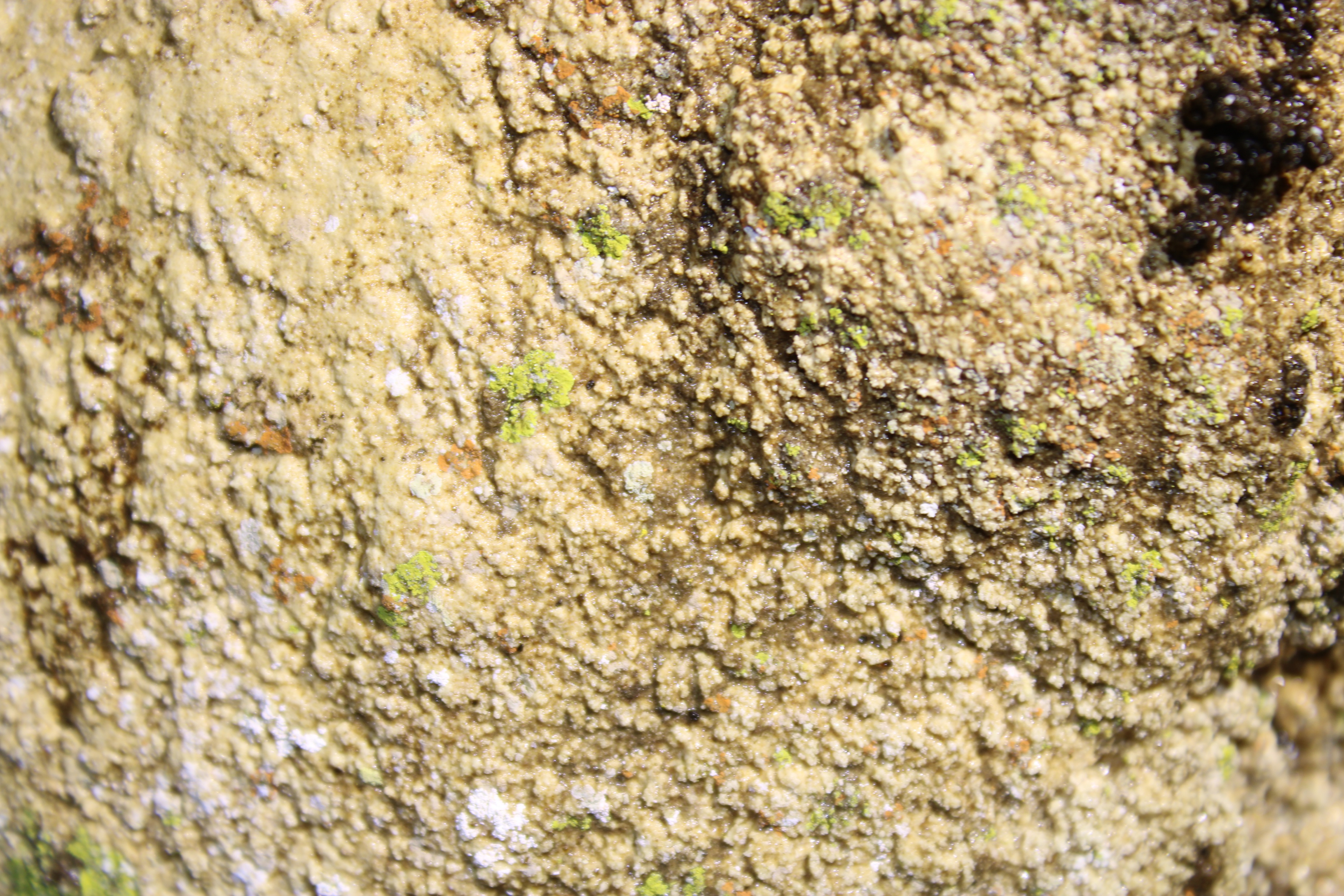

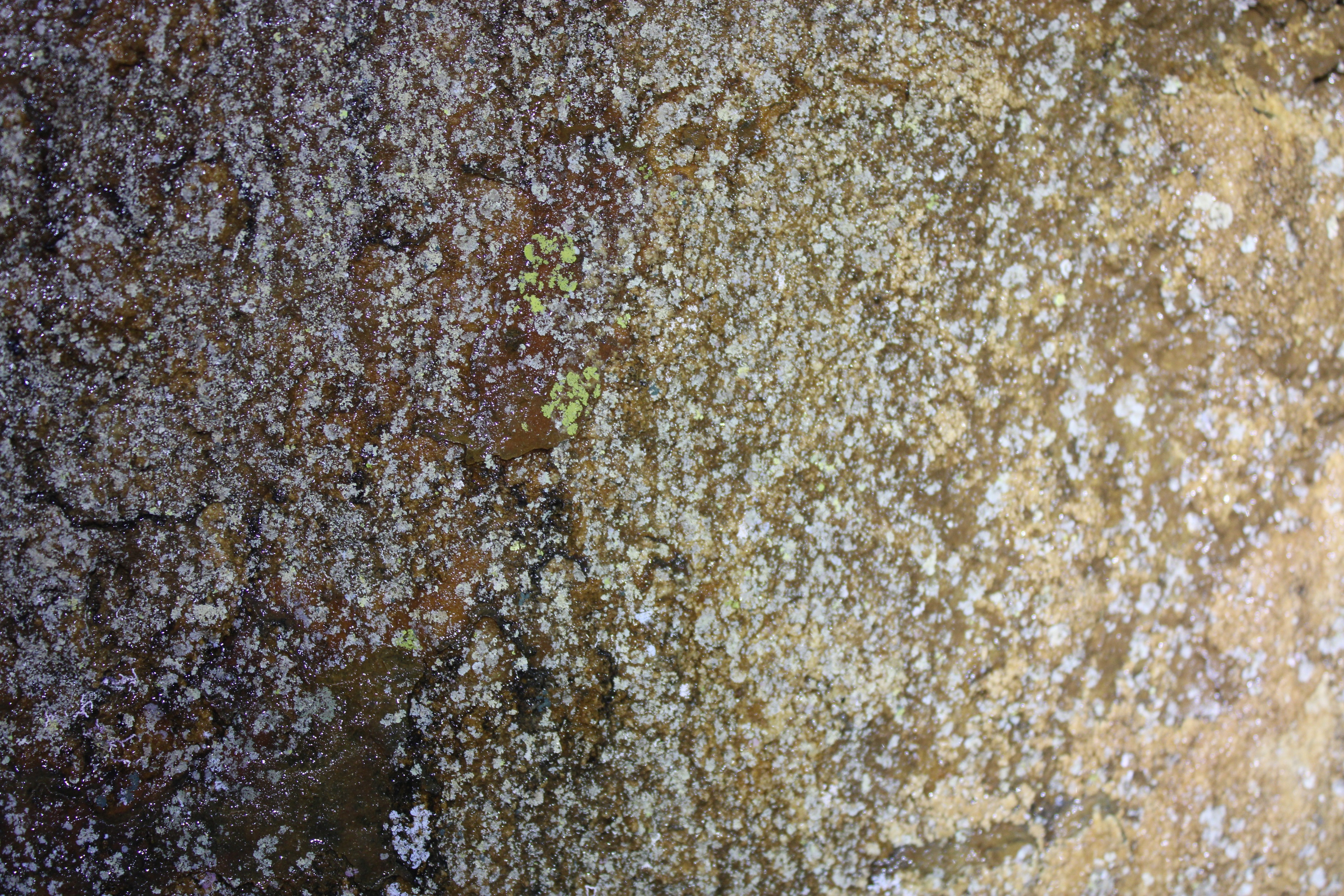

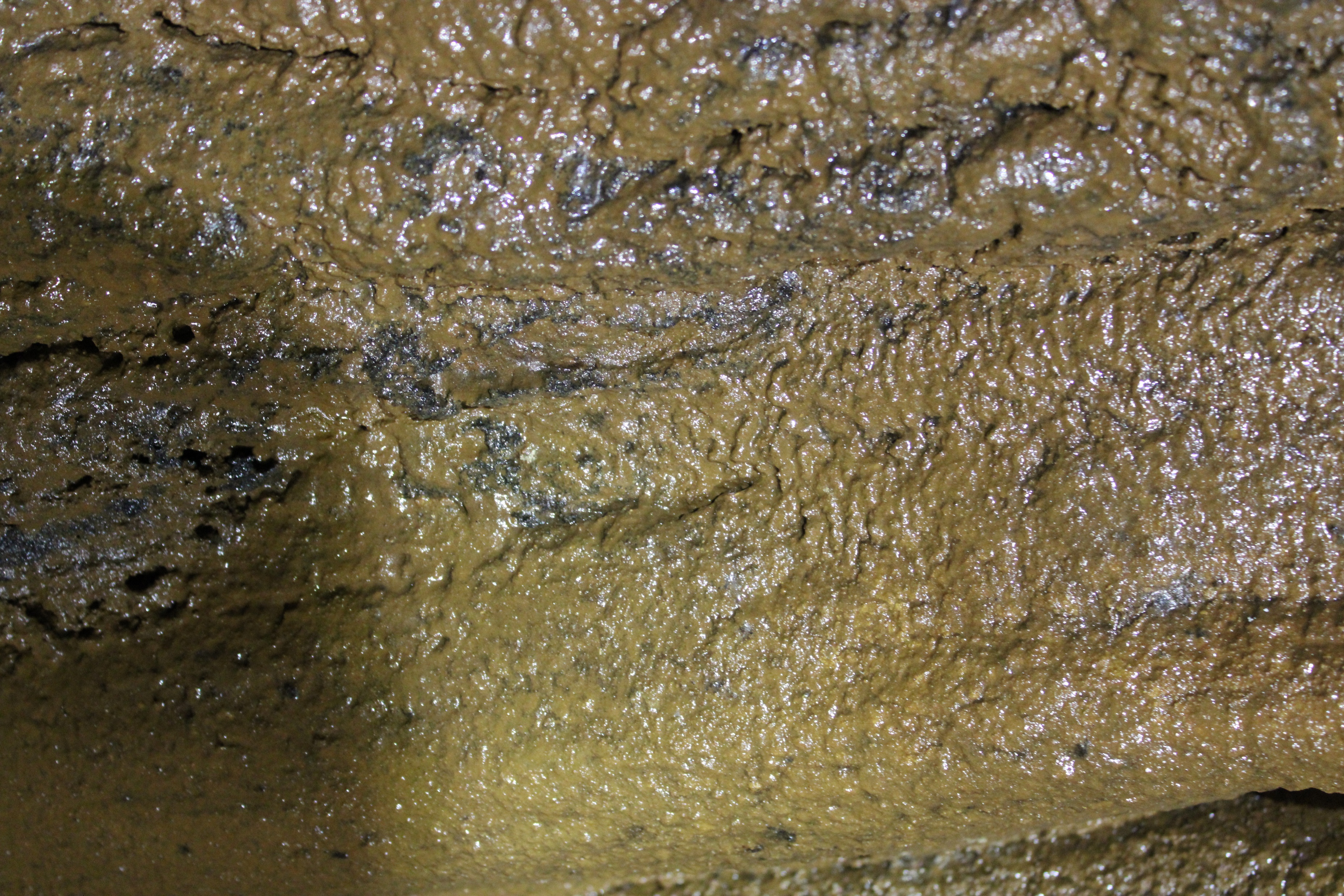

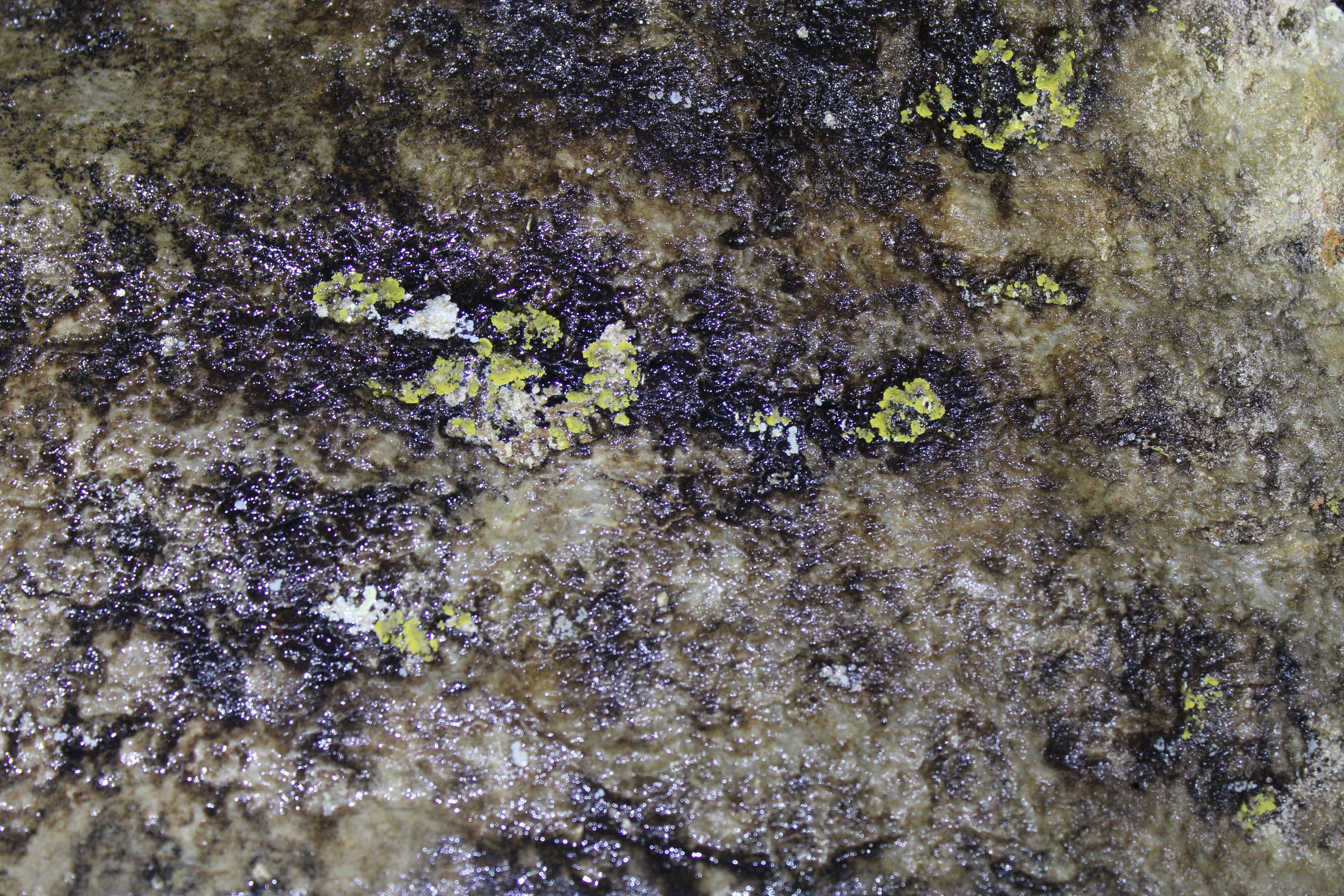

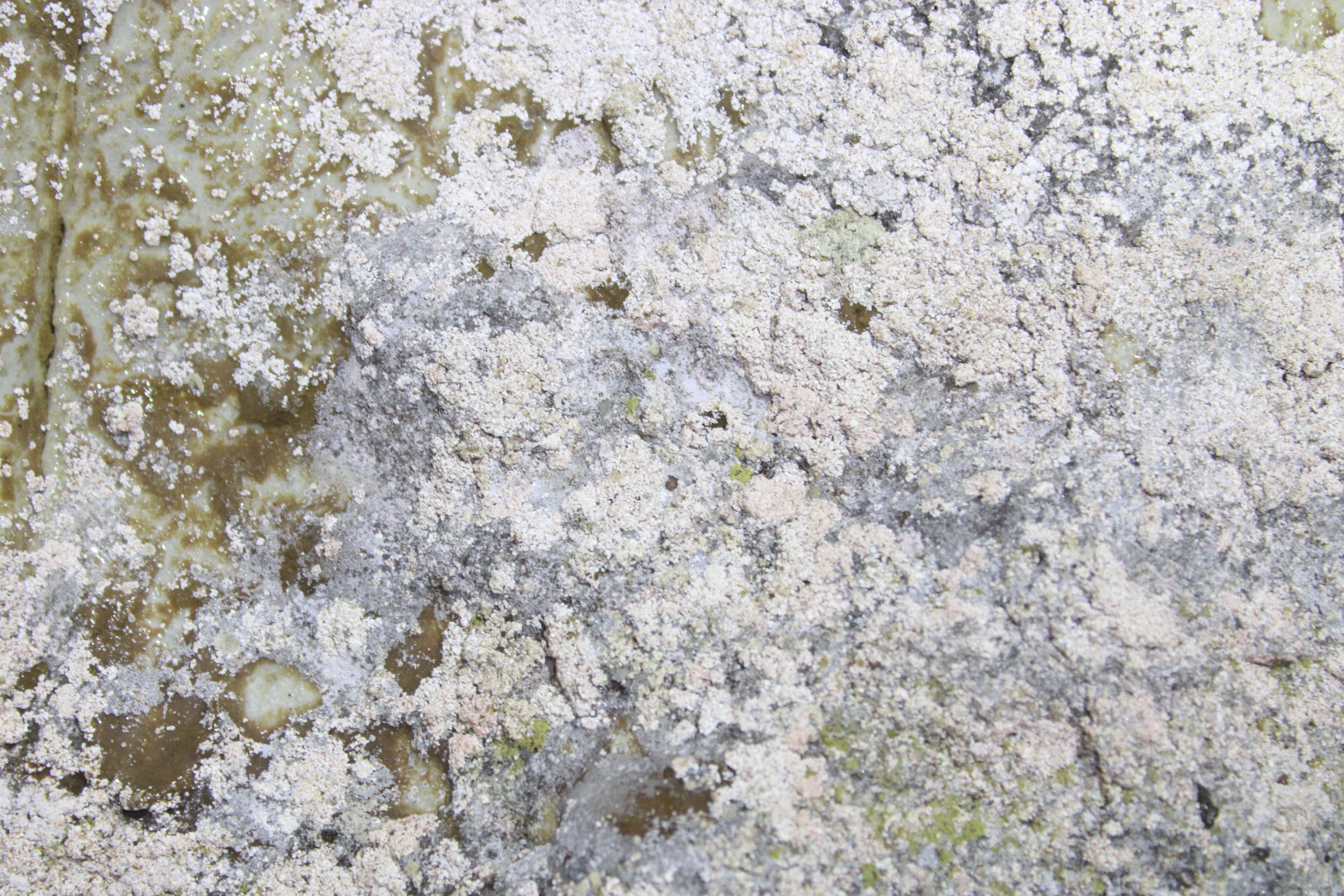

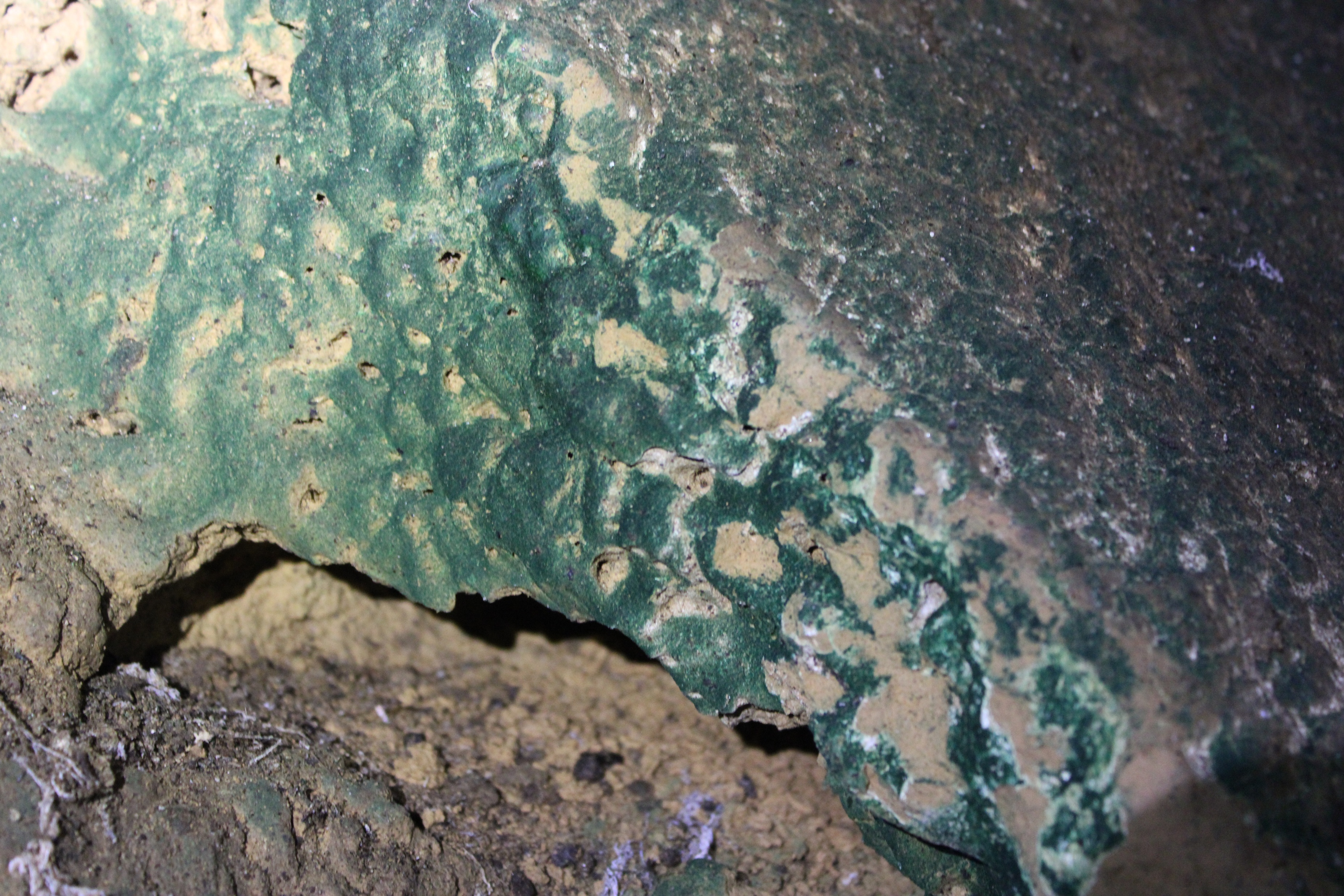

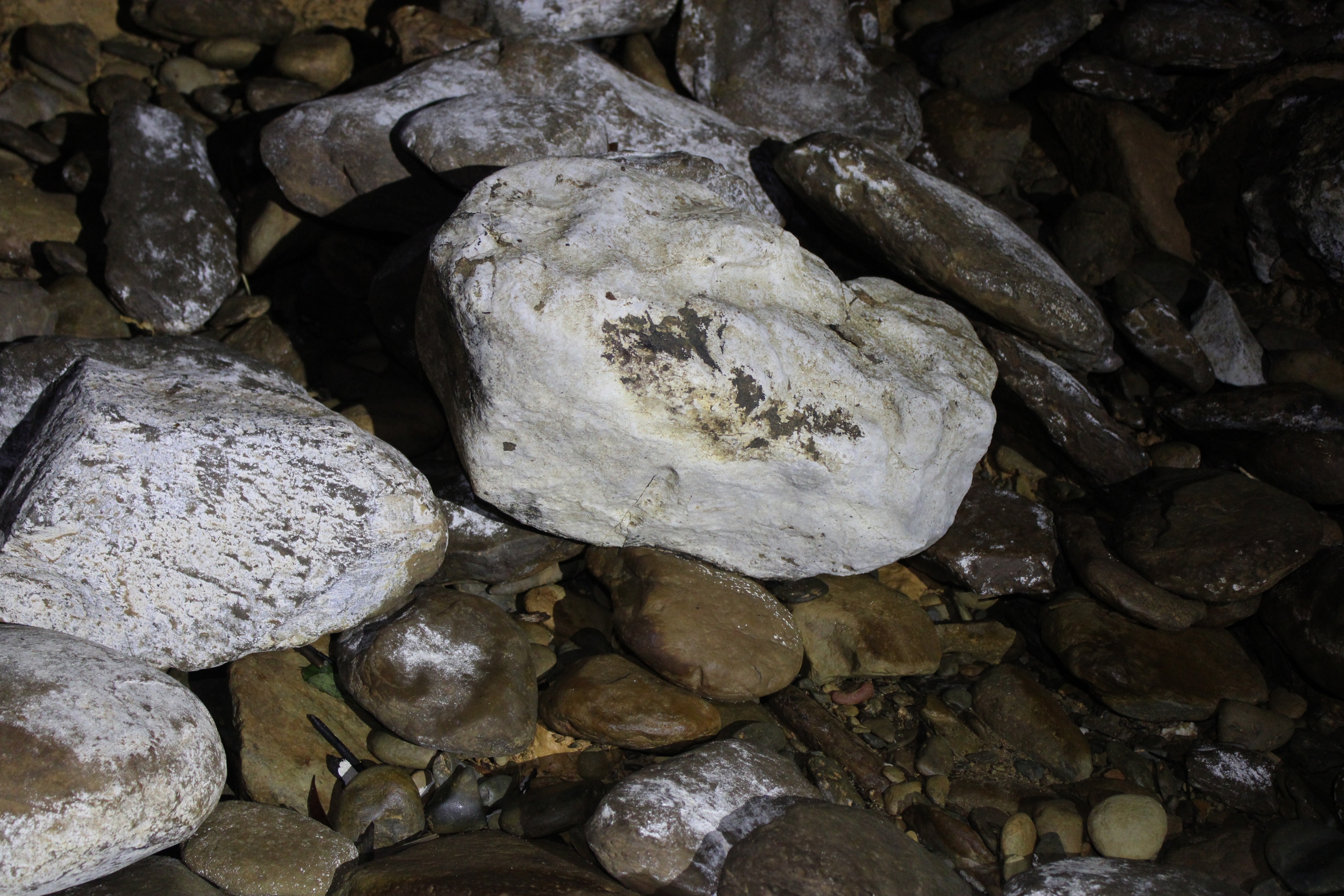


**30 cm**

**20 cm**

**a) D2**

**b) D3-W**

**c) D5**

**d) D6**

**e) L3-Y**

**f) L7-B**

**g) L13**

**h) L19**

**i) L21**

**j) L23**

**20 cm**

**15 cm**

**10 cm**

**10 cm**

**30 cm**

**10 cm**

**10 cm**

**50 cm**

Supplement: Supplementary file 1 — Supporting Information Additional supporting information can be found online in the Supporting Information section. Figure S1: Locations in (a) Deer Cave and (b) Lagang Cave where fresh biofilms were sampled for the isolation and screening of Actinobacteria for potential antibiotic production (cave map source: Department of Forestry, Sarawak). Figure S2: Biofilm samples collected from Deer Cave (D‐2, D‐3W, D‐5, and D‐6) and Lagang Cave (L‐3Y, L‐7, L‐13, L‐19, L‐21, and L‐23) within the Gunung Mulu National Park. Figure S3: Primary screening using the cross‐streak method. First, each actinobacterial isolate was streaked in the center of the plate. After 5 days of incubation at 28°C, the laboratory, hospital, and drug‐resistant strains of S. aureus and P. aeruginosa were streaked perpendicular to the grown actinobacterial isolates. The distance of inhibition on each side of the central streak was measured at the end of incubation. (b) Workflow after the submerged fermentation of the Actinobacteria. (c) Antimicrobial activity screening method of agar well diffusion using MHA plates with sample wells, negative control, and a commercial antibiotic disc as a positive control. Figure S4: Examples of media plates showing a zone of inhibition in negative control (NC) wells alongside the test samples. Although the agar well diameter was 8 mm, the negative controls (NCs) containing (a, b) ethyl acetate and (c) methanol showed slight inhibition of the test pathogens. Therefore, the “corrected zones of inhibition” reported in our study were calculated by subtracting the inhibition in the corresponding NC (mm) from that of the test sample (mm). [file IJM-2026-9984546-s001.zip › Figure S2.docx]

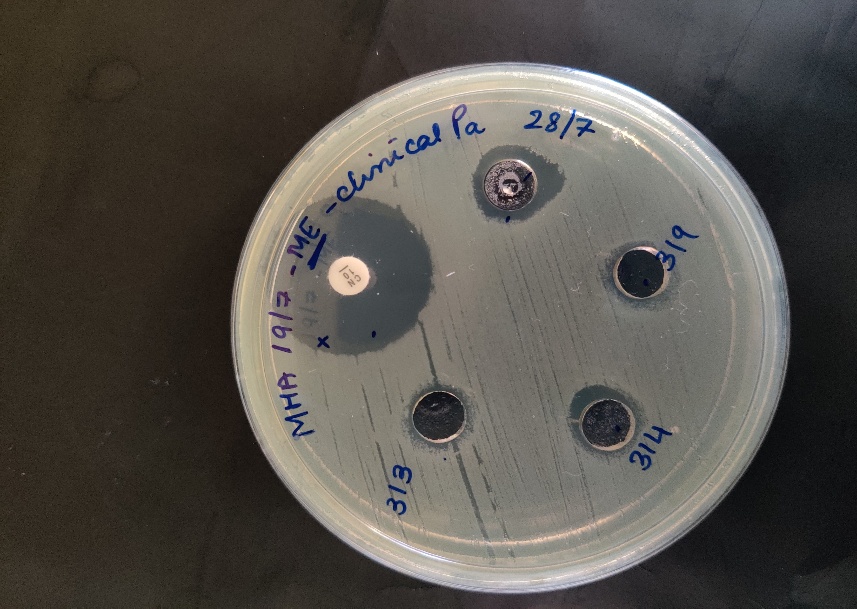

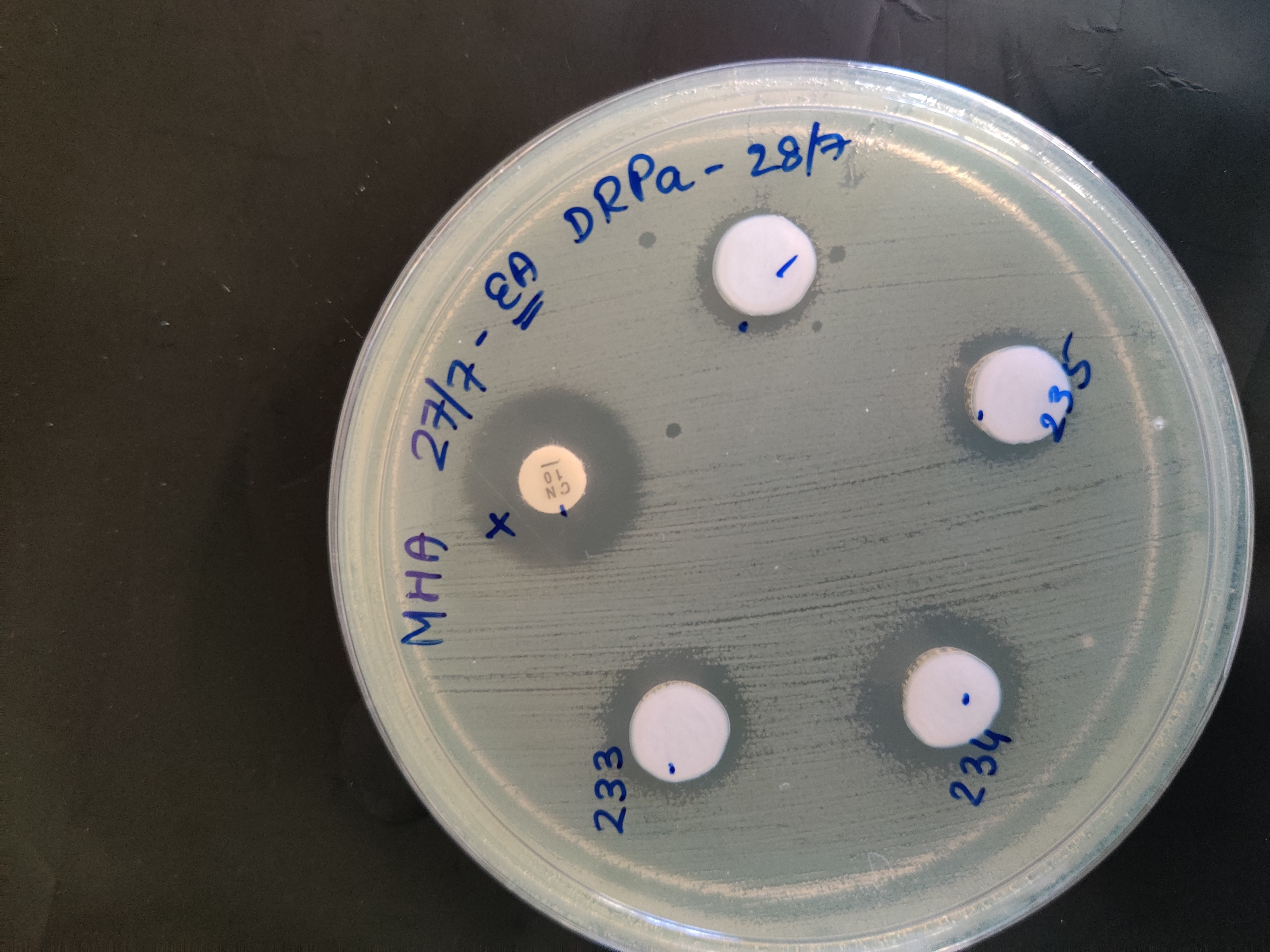

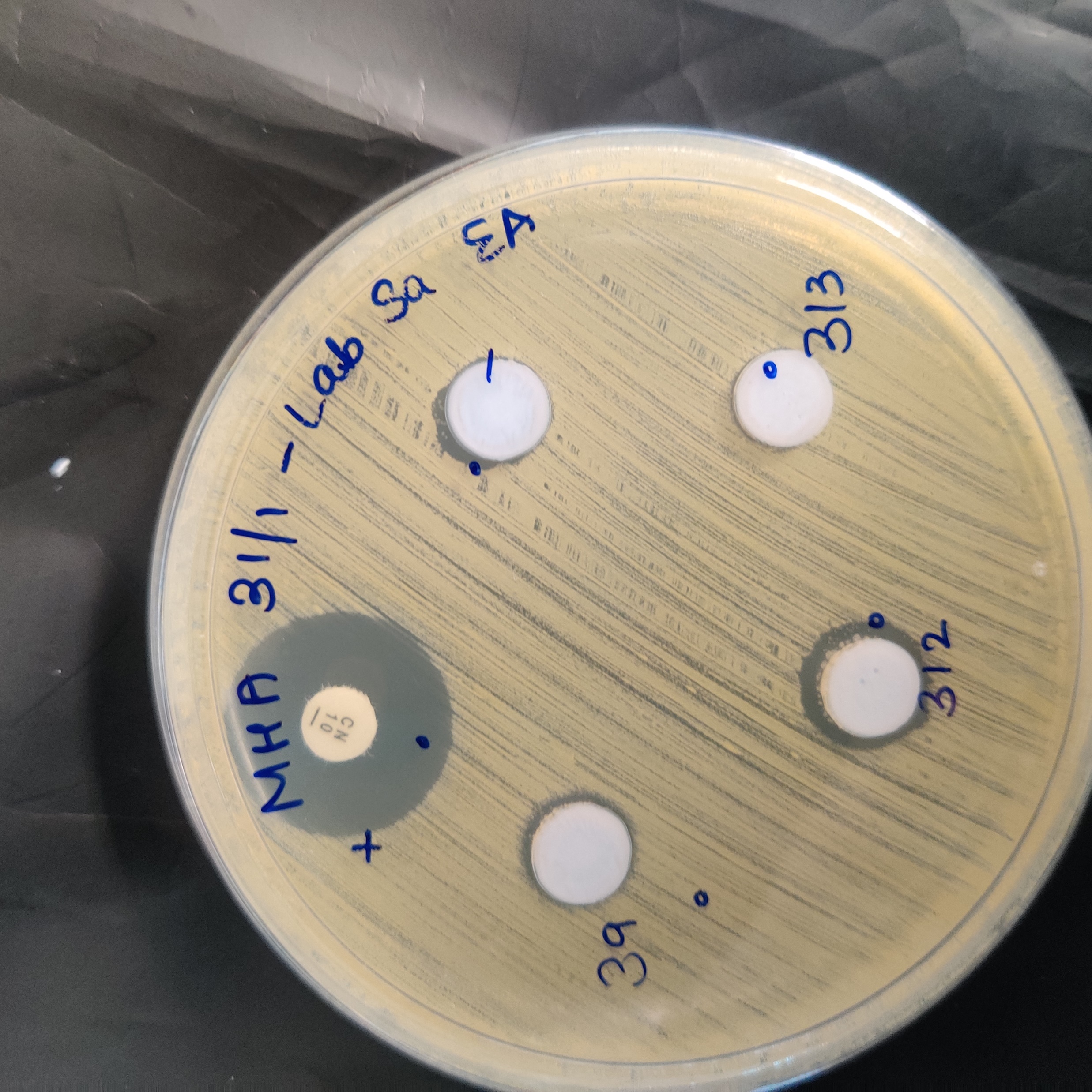

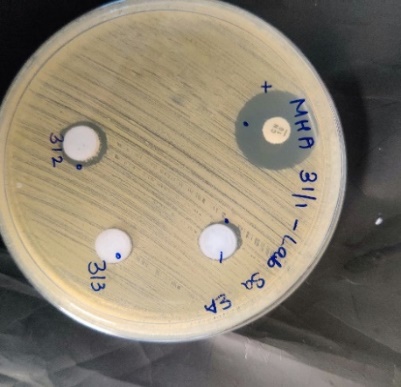


**a)**

**b)**

**c)**

**NC**

**NC**

**NC**

**PC**

**PC**

**PC**

Supplement: Supplementary file 1 — Supporting Information Additional supporting information can be found online in the Supporting Information section. Figure S1: Locations in (a) Deer Cave and (b) Lagang Cave where fresh biofilms were sampled for the isolation and screening of Actinobacteria for potential antibiotic production (cave map source: Department of Forestry, Sarawak). Figure S2: Biofilm samples collected from Deer Cave (D‐2, D‐3W, D‐5, and D‐6) and Lagang Cave (L‐3Y, L‐7, L‐13, L‐19, L‐21, and L‐23) within the Gunung Mulu National Park. Figure S3: Primary screening using the cross‐streak method. First, each actinobacterial isolate was streaked in the center of the plate. After 5 days of incubation at 28°C, the laboratory, hospital, and drug‐resistant strains of S. aureus and P. aeruginosa were streaked perpendicular to the grown actinobacterial isolates. The distance of inhibition on each side of the central streak was measured at the end of incubation. (b) Workflow after the submerged fermentation of the Actinobacteria. (c) Antimicrobial activity screening method of agar well diffusion using MHA plates with sample wells, negative control, and a commercial antibiotic disc as a positive control. Figure S4: Examples of media plates showing a zone of inhibition in negative control (NC) wells alongside the test samples. Although the agar well diameter was 8 mm, the negative controls (NCs) containing (a, b) ethyl acetate and (c) methanol showed slight inhibition of the test pathogens. Therefore, the “corrected zones of inhibition” reported in our study were calculated by subtracting the inhibition in the corresponding NC (mm) from that of the test sample (mm). [file IJM-2026-9984546-s001.zip › Figure S4.docx]
